# Physical Activity of Children with Visual Impairments during Different Segments of the School Day

**DOI:** 10.3390/ijerph17186897

**Published:** 2020-09-21

**Authors:** Jing Qi, Jian Wei Xu, Wei De Shao

**Affiliations:** College of Physical Education and Health Sciences, Zhejiang Normal University, Jinhua 321004, China; qijing@zjnu.edu.cn (J.Q.); 201925200572@zjnu.edu.cn (J.W.X.)

**Keywords:** physical activity, children with visual impairments, segments

## Abstract

Schools provide opportunities for children with visual impairments (VI) to accumulate recommended daily moderate-to-vigorous-intensity physical activity (MVPA). This study aimed to determine physical activity (PA) across the school day among special school children with VI in China. The study objectively measured the MVPA levels of children with VI during the recess, lunchtime, physical education (PE) classes, before-school, and after-school periods segments on PE days and non-PE days. Moreover, this research compared the gender, age, and body mass index (BMI) differences in MVPA during each segment. A total of 70 children with VI aged 7–17 years (mean age = 13.7; SD = 3.4) from the special school participated in this study. Accelerometers were utilized to measure the MVPA of children with VI. The participants with VI accumulated significantly more MVPA time on PE days than on non-PE days. Before-school periods and structured PE classes showed higher percentages of MVPA time than recess, lunch break, and after-school periods during the school day. Children with VI aged 7–12 years old were significantly more physically active than those aged 13–17 years old during recess, lunch break, and after-school periods. In conclusion, PA interventions during structured PE classes are recommended. Special attention should be provided to children with VI as they grow up to increase their MVPA.

## 1. Introduction

Regular participation in physical activity (PA) has physical and psychological benefits for all children, including those with disabilities [1,2]. The World Health Organization (WHO) [3] recommended that children aged 5–17 years participate in at least 60 min of moderate-to-vigorous-intensity physical activity (MVPA) daily. This recommendation for the general population is usually also applicable to children with disabilities [4]. However, children with visual impairment (VI) (i.e., blind or have low vision) do not meet the 60 min MVPA guidelines and tend to be more sedentary than their peers without disabilities [5,6,7,8,9,10] because of their lack of self-efficacy in performing PA [11,12], their decreased ability to perform daily tasks [13], and their lack of motivation to participate in PA [14,15,16]. Moreover, insufficient instruction and practice and overprotection prevent children with VI from engaging in PA [8,13].

Schools are important settings wherein children with VI engage in health-promoting PA because a considerable part of a child’s day is spent in school [17,18,19]. Similar to children without disabilities, children with VI have multiple opportunities, such as physical education (PE) classes, lunchtime, and recess, to be physically active during school time. Apart from school-based programs, after-school time is critical for children with VI to remain physically active through structured extracurricular activities, active travel, and recreational activities [20,21]. An improved understanding of how children with VI accumulate PA over the course of the day, which is segmented into the contributions of PE, lunchtime, recess, and after-school PA to overall school and daily PA patterns, is important to advance research on school activity promotion and integrated PA programming.

Several studies have examined the PA levels of children with disabilities during different school-day segments [9,22,23,24,25]. Pitetti et al. [24] used heart rate monitoring to assess the PA patterns of 15 grade 5 children with intellectual disabilities (Mean (M) age = 8.8 ± 2.2 years) during adapted PE, classroom, and recess in an inclusive elementary school in the USA. Their findings showed that PA during the adapted PE class represents the most important source of daily PA obtained during school hours, followed by classroom activities and recess. Li et al. [22] used an observational system to examine the PA levels of 35 children with physical disabilities (M age = 15.7 ± 4.3 years) in grades 4–12 from a special school during school (before school, recess, lunch break, and after class) and home (before dinner) time in Chinese Hong Kong. Their findings showed that the children were generally inactive in all settings but had slightly more PA during school recess and lunch break periods. Pan et al. [23] used accelerometers to compare the PA levels of 30 boys with autism spectrum disorders (ASD) with those of 30 boys without disabilities aged 12–17 years from inclusive secondary schools during school (PE, recess, and lunchtime) and after-school time in Taiwan. The results of this study demonstrated that participants with ASD spent less time engaging in MVPA than their peers without disabilities and that PA during after-school periods represented the most important source of daily PA during the school day, followed by PE, lunchtime, and recess. Sit et al. [9] also used accelerometers to examine the PA levels of children with diverse disabilities (M age = 13.04 ± 4.45 years) in three school settings (PE, recess, and lunchtime) in special schools in Chinese Hong Kong. The findings of this study showed that the three settings contributed significantly to MVPA during school time, with recess contributing more to MVPA than PE or lunchtime. Although studies have investigated the segmented PA of children with disabilities during school days, none have exclusively focused on that of children with VI. Moreover, some demographic characteristics, such as gender, age, and body mass index (BMI), have been widely reported to affect the segmented PA participation of children without disabilities [26,27,28]. However, how these factors influence the segmented PA of children with VI remains unknown.

In China, segregated special schools are main education placements and provide boarding facilities for children with disabilities. By 2018, China had 2152 special schools, which served 666,000 students with disabilities [29]. Special schools provide well-rounded educational programs that include adapted facilities and educational services that are specifically designed for individuals with VI, hearing impairments, and developmental disabilities. For example, the Nine-Year Compulsory Education Project for Blind Students’ School mandated that 6.3% of the total school curriculum time be allocated to PE and health [30]. The project also emphasized that schools should make overall arrangements for extracurricular PA programs and ensure that students with VI engage in physical exercises for least 1 h per day [30,31]. Wang (2019) [28] used accelerometers to examine MVPA of Chinese children without disabilities (M age = 9.28 ± 1.87 years) in grades 1–5 from three elementary schools during the recess, lunchtime, leisure time, PE, and physical exercise segments of the PE and physical exercise days. Leisure time was identified as the segment with the most MVPA time on PE days and physical exercise days. However, PE classes demonstrated the highest percentage time in MVPA among various segments. Despite a general awareness that school environments have the potential to contribute to the accumulation of the daily PA of children with VI, data on the PA levels of Chinese children with disabilities during school time are absent. Therefore, the present study focused on the MVPA levels of students with VI recruited from a special school and aimed to (1) measure their MVPA during different school-day segments, including PE, recess, lunchtime, before-school, and after-school periods, and (2) examine the influences of gender, age, and BMI on the PA levels of students with VI during different school-day segments. The analysis of segmented PA patterns can aid in obtaining a detailed understanding of the availability of PA opportunities for children with VI and developing, implementing, and evaluating interventions targeting the PA of such children [32,33].

## 2. Methods

### 2.1. Participants

We used a purposeful sample method and recruited 137 Chinese children with VI from a special school in Xingqing District in Yinchuan, a western city in China. The school services individuals with VI (*n* = 137) and hearing impairment (*n* = 132). This school includes primary school (grades 1–6, aged 7–12 years old) and middle school (grades 7–12, aged more than 13 years old). There are two classes per grade level. One class is for children with VI, and the other class is for children with hearing impairment. Each class comprises 5–15 students. Participants must meet the following criteria to be eligible for the present study: (a) 5–17 years of age; (b) diagnosed with low visual ability or blindness; (c) without other medical, intellectual, or physical disabilities; (d) present during the investigation, and (e) has valid accelerometer data covering at least three valid weekdays (at least one PE day). Of the 137 children with VI, 70 participants met the inclusion criteria. Therefore, the final analytic sample consisted of 70 participants (7–17 years; M age = 13.7, SD = 3.4), of whom 45 (63.4%) were males and 25 (35.7%) were females.

### 2.2. Research Setting

The special school is a residential school. The timetable of this school is arranged by the local education bureau. Students get up at 6:00 a.m. and go to sleep at 10:00 p.m. every day. The special school schedules the first class at 8:00 a.m. and the last class at 4:30 p.m. Thus, the during-school period is defined as 8:00 a.m. to 4:30 p.m., the before-school period is defined as between the waking time of the students and the beginning of the school day (i.e., 6:00 a.m. to 8:00 a.m.), and the after-school period is between the end of the school day and bedtime (i.e., 4:30 p.m. to 10:00 p.m.).

The main setting for PA engagement before school is 30 min (6:50 a.m. to 7:20 a.m.) of physical exercises in the morning. The participants of this study arrive at the school playground for morning physical exercises (e.g., running, walking, or rope skipping) at 6:50 a.m. with classroom teachers supervising activities to ensure safety.

Time segments during the school period were defined as lunch break, recess, and PE classes. Lunch break refers to 12:00 p.m. to 3:00 p.m. The school schedules lunch over a 30 min session (12:00 p.m. to 12:30 p.m.) in the cafeteria. Lunch is immediately followed by a 150 min midday nap in the dormitories. After the midday nap, students walk to their classrooms from the dormitories and are ready for class. Recess time refers to the break time between two classes. The recess periods are composed of the long break (15 min) and five segments that occur in conjunction with two classes. The total duration is 65 min. During the long break, students walk to the school grass soccer field and practice the Ninth Edition of the Broadcasting Gymnastics under supervision by their classroom teachers. The specific information related to the recess periods is shown in Table 1.

The special school schedules three 40 min PE lessons per week for students in the elementary school department and two 40 min PE lessons per week for students in the middle and high school departments. The school follows the specific PE curriculum for blind schools; this curriculum is one of main curricula and aims to promote the physical, psychological, and social adaptation of students while helping them develop a physically active and healthy lifestyle [31]. In this special school, a PE class is composed of the following routine sessions: (a) Lesson introduction by the PE teacher (approximately 5 min). Generally, the PE teachers emphasize safety knowledge in this session. (b) Warm up activities led by the PE teacher (approximately 5 min). (c) Physical activities (approximately 25 min). (d) Cool down and teaching conclusions (5 min). The core of each class is 25 min of teaching the Ninth Edition of the Broadcasting Gymnastics of China, running training by listening to whistling, practicing rope skipping and hula hooping, and a sports skill (e.g., learning goalball, passing a basketball, or volleyball serving). The PE teachers instruct students with VI to learn and practice these physical activities and sport skills. The school employs one female and four male PE teachers who range in experience from 7 years to 30 years. Amongst the five PE teachers, only two hold PE teacher education certificates. One is a Chinese teacher. The other two are administrative staff of the special school, and they teach PE as their part-time job. Facilities for PE lessons and recess in the school include one grass soccer field (outdoor), one activity area (outdoor), and a gymnasium.

During the after-school period (4:30 p.m. to 10:00 p.m.), most students need to take part in rope skipping practice for one hour. The special school in the current study implements Jump Rope Program for enhancing the physical and motor fitness of students with VI. This specialized program asks students with disabilities enrolled in this special school to practice jump rope during their after-school periods, and regularly trains those who have good motor skills for participation in National Games for Individuals with Disabilities. In addition, some students can participate in other sports training and specific group activities (e.g., chorus, performance class) organized by their school between 4:40 p.m. and 5:20 p.m. Students with low vision also need to help school workers clean their facilities (e.g., classrooms, bathrooms, halls, stairs, entrances, and gyms) every day during 5:20 p.m. to 6:00 p.m. Generally, students have their dinner at 6:30 (1 h) in the cafeteria. After dinner, students return to their classrooms to study or participate in some classroom-based activities led by their respective classroom teachers at school. Such activities include lectures on safety attention notice or a short meeting. The form and content of activities vary from class to class. Students return to their dormitories at 9:30 p.m. and go to sleep at 10:00 p.m.

### 2.3. Measures

**Background information.** The student affairs office of the school provided the background information, including gender, age, and grade of each participant. Age groups were defined. The participants were classified into two groups in accordance with the age division of primary and middle school of this special school and the WHO classification of age groups [3]. The age classifications were Age Group 1, which included students aged 7–12 years (*n* = 27) in the elementary school department, and Age Group 2, which included students aged 13–17 years (*n* = 43) in the middle and high school departments.

**Anthropometric measures.** During the school visits, one author and one graduate student measured the height and weight of each student. BMI was calculated by dividing weight (kg) by height (m) squared (kg·m^−2^). BMI groups were defined. The participants were classified into the normal-weight group (*n* = 49) and the abnormal-weight group (i.e., underweight, overweight, or obese) (*n* = 21) in accordance with the “Screening for Overweight and Obesity amongst School-age Children and Adolescents” [34].

**MVPA.** PA (as determined by counts/min) was measured for 7 days with ActiGraphGT3X accelerometers. This device has a high level of reliability and has been validated in youth with VI [6]. Each participant was instructed to wear the monitors for 24 h a day for eight consecutive days on his or her waist by using one of the compatible belts. The monitors may be retained during bathing or swimming due to their waterproof properties. The sampling interval (epoch) in the present study was set to 15 s; thus, accelerometer data were output in counts/15 s. After the test, the original ActiGraph data were processed with the ActiLife Lifestyle Monitoring System (software version 6.5.2, ActiGraph, Pensacola, FL, USA). A total of 600 min (10 h) of recorded accelerometer data per day (excluding strings of zeros for 20 min or longer) or more were considered valid [35]. At least three valid weekdays (including one PE day) of objective data were needed for inclusion in the final analysis of average PA daily time. MVPA was defined when the cut point was >3199 cpm in accordance with Puyau et al. [36]. The Puyau cut-off points are considered as the most suitable cut-off points for school-age children [37] and have been validated with children with VI [6,38]. Data were truncated and matched to the original time frames for each of the school segments daily.

### 2.4. Procedures

The protocol of the present study was approved by the Ethical Committee of the Zhejiang Normal University (kyy2020043). The principal and PE teachers of the school were contacted in advance to inform them of the project and to ask for the permission of school to conduct this study. After the school approvals were obtained, informed consent forms were distributed to the included students and their parents prior to data collection. Data were collected during the months of September 2018 to October 2018. One author and one graduate student majoring in sports pedagogy conducted the survey and test. The participants were asked to follow their normal daily routines during the monitoring period. Written instructions that reminded the students to wear the accelerometers were also provided to the teachers in charge of the classes of the participants to increase compliance. Participants were required to return the accelerometers after data collection.

### 2.5. Statistical Analyses

M, standard deviations (*SD*), and percentages (MVPA time divided by the total time of each school segment) were used to obtain MVPA time during the school-day segments. Multiple 2 × 2 × 2 (gender × age group × BMI group) analysis of variance (ANOVA) tests were utilized to analyze the effect of gender, age, and BMI on MVPA during the before-school period, PE classes, recess, lunch break, and after-school period. Eta-squared (η^2^) was used to provide estimates of effect size (ES) [39,40]. The following values that were outlined by Cohen [41] were used to define ES magnitude: 0.01–0.06 = small effect, 0.06–0.14 = medium effect, and >0.14 = large effect. Significance level was considered at *p* < 0.05. SPSS 22.0 (IBM, Armonk, NY, USA) was used for data analysis.

## 3. Results

### 3.1. MVPA Time on PE and Non-PE Days

Overall, the participants with VI in this study engaged in an average of 168.62 min of MVPA for an entire school day. A total of 98.6% of the participants met the recommendations of 60 min of daily MVPA. Paired *t*-test results demonstrated that children with VI accumulated significantly more MVPA time on PE days (M = 181.18 min, *SD* = 73.68) than on non-PE days (M = 160.57 min, *SD* = 71.05, *t*(1,69) = 4.712, *p* < 0.001).

### 3.2. MVPA Time and Gender, Age, and BMI Differencs during Different Segments

**Figure 1:**
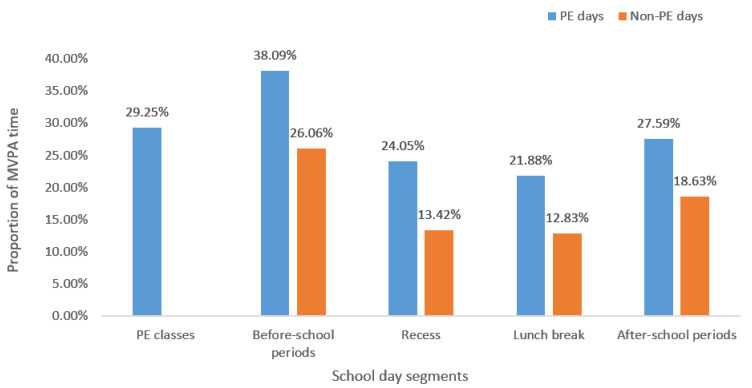
Proportion of moderate-to-vigorous physical activity (MVPA) time during different segments of physical education (PE) and non-PE days.

The MVPA percentage during each school segment on PE and non-PE days (Figure 1), and differences in MVPA by age, gender and BMI were presented in the following. Table 2 shows the Ms, SDs, and F-values for MVPA times during school-day segments. Three-way ANOVA (gender × age group × BMI group) was conducted separately for each individual segment.

#### 3.2.1. PE Classes

On PE days, the children with VI spent an average of 11.70 min (*SD* = 6.82) on MVPA during the PE classes, and this accounted for 29.25% of class time. No significant age (*F*(1,69) = 2.972; *p* = 0.089; η^2^ = 0.042), gender (*F*(1,69) = 2.170; *p* = 0.145; η^2^ = 0.031), and BMI (*F*(1,69) = 1.622; *p* = 0.207; η^2^ = 0.023) differences in MVPA time were observed during PE classes.

#### 3.2.2. Before-School Periods

The children with VI spent 38.09% and 26.06% of the before-school periods performing MVPA on PE and non-PE days. Paired *t*-test results demonstrated that children with VI accumulated significantly more MVPA time during the before-school periods on PE days (M = 45.71 min, *SD* = 13.79) than on non-PE days (M = 31.27 min, *SD* = 12.59, *t*(1,69) = 15.277, *p* < 0.001). No significant age, gender, and BMI differences in MVPA time were observed during the before-school periods on PE and non-PE days.

#### 3.2.3. Recess

The children with VI spent 24.05% and 13.42% of recess engaging in MVPA on PE and non-PE days. Paired *t*-test results demonstrated that children with VI accumulated significantly more MVPA time during recess on PE days (M = 15.63 min, *SD* = 10.27) than on non-PE days (M = 8.72 min, *SD* = 5.89, *t*(1,69) = 7.141, *p* < 0.001). No significant age, gender, and BMI differences in MVPA time were observed during recess on PE and non-PE days. However, the results showed that age had significant main effects on the MVPA time of children with VI during recess (*F*(1,69) = 4.646; *p* = 0.035; η^2^ = 0.064) on PE days. Participants aged 7–12 years engaged in more MVPA time during recess than those aged 13–17 years old on PE days (*F*(1,69) = 4.646; *p* = 0.035; η^2^ = 0.064).

#### 3.2.4. Lunch Break

The children with VI spent 21.89% and 12.83% of lunch break engaging in MVPA on PE and non-PE days. Paired *t*-test results demonstrated that children with VI accumulated significantly more MVPA time during lunch break on PE days (M = 39.38 min, *SD* = 20.81) than on non-PE days (M = 23.10 min, *SD* = 12.67, *t*(1,69) = 9.807, *p* < 0.001). No significant gender and BMI differences in MVPA time were observed during lunch on PE and non-PE days. The results showed that age had significant main effects on the MVPA time of children with VI during lunch break. Participants aged 7–12 years engaged in more MVPA time during lunch break than those aged 13–17 years old on PE (*F*(1,69) = 12.706; *p* = 0.001; η^2^ = 0.157) and non-PE days (*F*(1,69) = 9.083; *p* = 0.004; η^2^ = 0.118).

#### 3.2.5. After-School Periods

The children with VI spent 27.59% and 18.63% of the after-school periods engaging in MVPA on PE and non-PE days. Paired *t*-test results demonstrated that children with VI accumulated significantly more MVPA time during the after-school periods on PE days (M = 91.06 min, *SD* = 37.63) than on non-PE days (M = 61.48 min, *SD* = 31.58, *t*(1,69) = 10.958, *p* < 0.001). No significant gender and BMI differences in MVPA time were observed during the after-school periods on PE and non-PE days. The results showed that age had significant main effects on the MVPA time of children with VI during the after-school periods. Participants aged 7–12 years engaged in more MVPA time during the after-school periods than those aged 13–17 years old on PE (*F*(1,69) = 9.295; *p* = 0.003; η^2^ = 0.120) and non-PE days (*F*(1,69) = 12.805; *p* = 0.001; η^2^ = 0.158).

## 4. Discussions

The results of the current study showed that children with VI spent an average of 168.62 min on MVPA daily. This value is well above the recommended level. Thus, within this school’s environment, children with VI had sufficient PA participation during the school day. This finding is inconsistent with the results of previous studies that found that children with VI [5,6,8,9,10] and children without disabilities [28] fail to reach the recommended 60 min/day of MVPA. Four reasons might explain the high MVPA of children with VI found in the current study. Firstly, Government policies support and promote healthy active lifestyles among children with disabilities. With China’s considerable economic development, the Chinese government has paid increasing attention to the physical and psychological health of children and adolescents, including those with disabilities. At the policy level, the Plan for Healthy China 2030 [42] and the Plan for Individuals with Disabilities to Live Decent Lives [43] proposed various strategies to promote the quality of life of individuals with disabilities. Accordingly, the special school in this study provides various opportunities to students with VI to engage in physical activities. Secondly, when compared with children without disabilities in general schools who need to travel to school, children with VI in the current study possessed more time and opportunities to engage in MVPA, including doing half an hour of morning physical exercises every day and participating in some physical activities during their three-hour lunch break. In addition, children with VI in the current study were arranged to participate in rope skipping practice for one hour during the after-school periods. It appeared that expanded school-based PA opportunities significantly helped children with VI meet and/or exceed PA guidelines. Thirdly, compared with general schools, Chinese special schools pay more attention to the social and intelligence development of students with disabilities through training the students in everyday living skills and physical improvement and career specialties rather than focusing on their academic performance [44]. Therefore, students with VI are not under high academic pressure; subsequently, they do not need to devote considerable time to academic learning, thus assuring sufficient time for engagement in physical activities. The fourth reason for the high level of the PA participation of students with VI may be related to the emphasis on daily activities amongst individuals with VI. Previous data indicated that the duration, level, and cause of VI do not influence sports and PA participation [45]. Children with VI may not experience their disability as a barrier to engaging in PA participation.

This study found that children with VI were physically more active on PE days than on non-PE school days. This finding was consistent with previous studies [46,47]. It highlighted that PE has a positive contribution on MVPA levels of children with VI during the school days. Moreover, the results of the study also showed that children with VI were physically more active during other segments (i.e., before-class, recess, lunch break, and after-class) on PE days than during the same settings on non-PE days. Based on the trans-contextual model of motivation [48], previous research [49,50] has demonstrated that elements of the PE lesson at school, such as teaching style, and pedagogical methods and practices can enhance motivation of PA participation among children without disabilities during other time periods on the same day [51], which may promote PA levels of children with VI during other school segments in the current study.

Scrutinizing the school-based PA programs showed that they yielded different relative percentage times in MVPA. Specifically, in the present study, the highest percentage time in MVPA was obtained during before-school periods. This finding was not surprising because students with VI routinely arrive at the playground and start their school day with walking, jogging, gymnastics, or a series of stretching and movement exercises for half an hour in the morning every day. These activities might account for the highest MVPA percentage obtained in this work.

The lowest percentage time in MVPA was obtained during lunch break. This finding was consistent with the results of previous studies [9,22], which reported that lunch break showed the lowest percentage time in MVPA of children with disabilities during the school day. This finding was also consistent with Wang (2019) [28], who reported that lunch break showed the lowest percentage of MVPA time among the general school segments in Chinese children without disabilities. Asian countries, such as China and Singapore, have traditions that suggest that school-aged children should nap during lunch break [52]. Children with VI in the current study napped for approximately 2 h/day on average during lunch break. This practice might account for the lowest MVPA time obtained during this period.

Notably, PE classes did not demonstrate the highest percentage time in MVPA in the current study. This finding is inconsistent with the results of previous studies, which found that amongst children without disabilities [28,53,54] and children with disabilities [9,23,25], the average percentage of time spent in MVPA during PE was substantially higher than that during other school segments. Students with VI in the current study spent approximately 29.25% of PE class time engaging in MVPA and did not meet the 50% of PE time criterion [55,56]. Moreover, this finding was lower than that presented in review articles [57,58], which reported that the MVPA value of students without disabilities in elementary, middle, and high school is between 34.2% and 47% of PE class time. This shortfall may reflect the educational characteristics of PE, which focuses on using physical activities as a vehicle for learning in different domains, such as social, motor, cognitive, and affective development. In a typical PE lesson, students are required to stop their activity at regular intervals to receive instructions, observe demonstrations, or organize equipment and groupings. In particular, students with VI require additional instruction and practice time to learn new concepts and movements [59,60]. These pedagogical episodes make improving and maintaining high levels of MVPA in PE challenging for teachers [57]. Therefore, developing intervention strategies to meet the MVPA levels of students with VI during PE classes is necessary.

The MVPA percentage during recess was lower than that during the before-school periods and PE classes. This finding was consistent with Wang (2019) [28], who reported that Chinese children without disabilities engaged in lower MVPA during recess. The recess interval was short, and the children with VI in the current study may prefer to sit, sleep, or talk to peers during recess, which may result in less PA participation. The MVPA percentage during the after-school periods was also lower than that during the before-school periods and PE classes. This finding was expected because after-school time in the current study (i.e., 330 min) is the longest segment.

The results of the present study indicated that younger participants with VI were significantly more physically active than older participants during the majority of school segments on PE or non-PE days. The age-related declines in PA observed in this work were consistent with the results of research on children with [5,8,61,62,63,64] and without disabilities [65,66]. Increasing homework or participation in organized activities during the after-school periods from children to adolescents is likely responsible for decreases in PA. In addition, in the setting of the current study, PE is mandatory for three times a week in the elementary school department and for two times a week in the middle and high school department. One less PE class might have resulted in reduced PA participation amongst older children with VI in the current study. Transition planning for students with VI to facilitate active PA participation as they grow is warranted [67]. Future research should carefully investigate the reasons for age-related declines in PA among children with VI.

### Strengths and Limitations

This study used accelerometers to measure MVPA of children with VI during the different segments of school days. Thus, it provided comparative data that would allow researchers and practitioners to learn specific information about the MVPA levels of children with VI during school days and develop PA interventions. In addition, this study enhanced the analysis of the PA patterns of Chinese children with VI throughout different school segments by discussing the differences contributed by gender, age, and BMI. The limitations of this study were as follows: (a) The findings were based on a small group of children with VI (i.e., 70 children with VI) from one special school. Hence, the results may not be generalized to other children with VI from other schools. A large sample of multiple schools should be targeted in future studies. (b) This research did not examine the influences of VI levels on MVPA levels during different school segments because of limited data. Therefore, whether the high levels of the MVPA of Chinese children with VI in this study were related to VI levels is unknown. The use of standardized VI classification systems should be considered when conducting future research [68]. (c) Qualitative data were not collected on PA, which could have helped identify the types of PA in which children with VI engaged during each school segment.

## 5. Conclusions

This study provided a detailed breakdown of the PA levels of children with VI in China during the school day. Almost all children with VI met the recommended PA at school. Specifically, children with VI accumulated significantly more MVPA time on PE days than on non-PE days. Before-school periods and structured PE classes showed higher percentages of MVPA time than recess, lunch break, and after-school periods during the school day. Children with VI aged 7–12 years old were significantly more physically active than those aged 13–17 years old during recess, lunch break, and after-school periods. Special attention should be provided to children with VI as they grow up to increase their MVPA.

### Implications

This study’s results suggest that the residential special school in the current study offers an optimal environment to increase PA of children with VI; especially segments such as before- and after-school periods are important sources for MVPA accumulation of children with VI. Therefore, it should be considered for policies, timetables, and curriculums in order to offer sufficient opportunities for children with VI to be physically active during school periods. In addition, in accordance with the research conclusion, the reduced level of activity in children with VI during PE lessons suggests that strategies for enhancing the PA of children with VI during PE classes should be redesigned and developed. For example, preteaching is one of the useful instructional strategies for providing students with VI with sufficient opportunities to increase their PA engagement during actual PE classes. Preteaching may include tactile maps, orientation, and mobility practice and actual instruction in and practice of foundational concepts and skills [69]. Moreover, equipping qualified PE teachers with a background or previous experience in providing accommodations or modifications to students with VI is crucial for increasing the MVPA levels of students in structured PA programs at schools. Additional focus may be necessary during adolescence given that the current study found that increasing age is an important negative factor that affected PA during different school segments. Given the current policy of 2 days of PE per week in middle and high school in Chinese special schools, additional opportunities to promote PA for children with VI appear urgent.

## Figures and Tables

**Table 1:** Recess periods of the special school.

	Time	Min	Main Contents
1	8:20–8:30	10	Between morning meeting and the first class
2	9:10–9:20	10	Between the first and second classes
3	10:05–10:20	15	Long break
4	11:00–11:10	10	Between the third and the fourth classes
5	11:50–12:00	10	Between the fourth class and lunch
6	15:40–15:50	10	Between the fifth and the sixth classes
	Total	65	―――

**Table 2:** Gender, age, and body mass index (BMI) differences in MVPA on PE and non-PE days.

School-Day Segments		Gender		*F*	Age		*F*	BMI		*F*
		Boys (*n* = 45) M (*SD*)	Girls (*n* = 25) M (*SD*)		7–12 Years Old (*n* = 27) M (*SD*)	13–17 Years Old (*n* = 43) M (*SD*)		Normal Weight (*n* = 49) M (*SD*)	Abnormal Weight (*n* = 21) M (*SD*)	
MVPA on PE days	PE class	12.59 (7.43)	10.10 (5.33)	2.170	9.95 (6.30)	12.80 (6.98)	2.972	12.38 (6.90)	10.12 (6.52)	1.622
	Before-school periods	46.14 (13.77)	44.93 (14.07)	0.120	46.19 (15.64)	45.40 (12.68)	0.054	47.23 (13.16)	42.15 (14.87)	2.027
	Recess	14.49 (9.15)	17.70 (11.96)	1.579	17.68 (9.38)	12.38 (10.95)	4.646 *	15.80 (11.27)	15.24 (7.66)	0.043
	Lunch break	35.91 (19.20)	45.62 (22.50)	3.628	49.72 (22.67)	32.88 (16.77)	12.706 **	38.88 (20.90)	40.54 (21.05)	0.092
	After-school periods	86.73 (39.37)	98.86 (33.63)	1.687	107.41 (41.99)	80.80 (90.90)	9.295 **	93.38 (39.93)	85.68 (31.85)	0.609
MVPA on non-PE days	Before-school periods	30.82 (13.10)	32.08 (11.82)	0.159	34.35 (13.65)	29.33 (11.62)	2.700	31.22 (11.69)	31.37 (14.78)	0.002
	Recess	8.73 (5.34)	8.71 (6.89)	0.000	7.51 (5.80)	9.49 (5.88)	1.894	8.73 (5.89)	8.71 (6.03)	0.000
	Lunch break	21.43 (13.28)	26.10 (11.13)	2.225	28.55 (14.57)	19.68 (10.07)	9.083 **	23.16 (12.29)	22.97 (13.83)	0.003
	After-school periods	61.98 (34.60)	60.59 (25.93)	0.031	77.24 (36.28)	51.59 (23.76)	12.805 **	62.70 (31.85)	58.65 (31.54)	0.239

*Note:* * *p* < 0.05, ** *p* < 0.01. M: mean. *SD*: standard deviation; MVPA: moderate-to-vigorous-intensity physical activity; PE: physical education; BMI: body mass index.
